# Histopathological and immunohistochemical characteristics of chordae tendineae affected by degenerative processes in canine myxomatous mitral valve disease

**DOI:** 10.1007/s11259-025-10761-5

**Published:** 2025-05-06

**Authors:** Justyn Gach, Agnieszka Mackiewicz, Izabela Janus-Ziółkowska, Agnieszka Noszczyk-Nowak

**Affiliations:** 1 Department of Internal Medicine and Clinic of Diseases of Horses, Dogs and Cats, Faculty of Veterinary Medicine, Wrocław University of Environmental and Life Sciences, Grunwaldzki sq. 47, Wrocław, 50-366 Poland; 2 Department of Biomedical Engineering, University of Zielona Góra, Prof. Z. Szafrana 4 Street, Zielona Góra, 65-516 Poland; 3 Department of Pathology, Faculty of Veterinary Medicine, Wrocław University of Environmental and Life Sciences, C. K. Norwida 31 Street, Wrocław, 50-375 Poland

**Keywords:** Canine, Chordae tendineae, Histopathology, Immunochemistry, Myxomatous mitral valve disease

## Abstract

Myxomatous mitral valve disease is a major problem in canine cardiology. Degenerative changes extend from the valve leaflets to reach the rest of the subvalvular apparatus. The chordae tendineae (CT) play a key role in the mechanics of the mitral valve and ensure unidirectional blood flow through the heart. Degenerative changes within the chordae tendineae can severely disrupt their function, ultimately leading to an episode of chordae rupture. The study aimed to analyse the structure of healthy and degenerated CTs via histopathology and immunohistochemistry. The mitral valve was assessed macroscopically using the Whitney scale to identify degenerative changes. The chordae tendineae were classified on a four-grade scale (0–3) on the basis of structural changes and subsequently analysed through immunohistochemical staining with antibodies targeting collagens I, III, and IV, as well as fibronectin, chondroitin, and tenascin. The findings revealed alterations in the extracellular matrix in degenerated chordae tendineae.

## Introduction

Myxomatous mitral valve disease (MMVD) is a serious issue in veterinary cardiology and is responsible for approximately 75% of cases of canine heart diseases (Häggström et al. 2004; Borgarelli and Haggstrom 2010). The presence of degenerative changes contributes to valvular regurgitation and can ultimately result in heart failure, leading to increased mortality in approximately 30% of canine MMVD cases (Borgarelli and Haggstrom 2010). It is the most common cause of cardiac-related mortality in dogs (Häggström et al. 2009). The mitral valve is a complex structure consisting of an annulus, two leaflets — septal and parietal — chordae tendineae and two papillary muscles (Frater and Ellis 1961). Its proper function is essential for maintaining unidirectional blood flow through the heart. The chordae tendineae (CT) are fibrous cords that connect the papillary muscles to the atrioventricular valves. During ventricular systole, contraction of the papillary muscles generates tension in the CT, preventing prolapse of the leaflets into the atria. This mechanism is crucial for maintaining normal valve function and overall cardiac performance (Karas and Elkins 1970).

Degenerative changes within the mitral valve include macroscopic and microscopic changes. Nodule-like lesions appear on the valve and progress with greater disease severity (Whitney 1974). Collagenous and most non-collagenous components are reduced in myxomatous areas, whereas the expression of glycosaminoglycans (GAGs) is elevated (Hadian et al. 2007; Han et al. 2010). The degenerative alterations encompass not only the mitral valve leaflets but also the CTs (Jiranantasak et al. 2014). The normal CTs are composed of connective tissue elements, including collagen fibrils, elastic fibres, glycoproteins, and proteoglycans (Scott-Jupp et al. 1981). Collagen fibres are highly organized and aligned with the direction of the load to endure the high tensile stress exerted on them (Grande-Allen 2004). Throughout the progression of degenerative mitral valve disease and CT pathology, alterations in extracellular matrix components occur, leading to compromised biomechanical integrity (Akhtar et al. 1999). In dogs and humans, these pathological changes are characterized by a reduction in collagen, collagen fragmentation, an increase in the GAG concentration, modifications in GAG composition, as well as progressive swelling, thickening, and ultimately rupture (Buchanan 1977; Baker et al. 1988). Rupture of the CT can precipitate acute mitral regurgitation, leading to rapid clinical deterioration. This condition often manifests as sudden-onset heart failure and signs of pulmonary edema (Serres et al. 2007; Boudoulas et al. 2023). Despite the significant advancements in understanding degenerative mitral valve disease in dogs, information on changes in the chordae of this species is scarce (Fox 2012; Markby et al. 2017). In recent years, significant attention has been given to elucidating the pathobiology of valvular degenerative disease, driven by advancements in veterinary molecular biology, as well as detailed histopathological and immunohistochemical studies (Markby et al. 2017).

Chordae tendineae in dogs are classified into 1^st−^ and 2nd -order CTs on the basis of their structural and functional roles in the heart. First-order CTs attach directly to the free margins of the valve leaflets and play a crucial role in preventing valve prolapse during systole. Second-order CTs connect to the ventricular surface of the leaflets or their basal regions, providing additional structural support and distributing mechanical tension more evenly across the valve apparatus (Fox 2012). A characteristic feature consistently associated with MMVD is the elongation and/or rupture of the CT. This phenomenon may play a role in the disease progression from mild valve prolapse to severe regurgitation (Baker et al. 1988; Serres et al. 2007). It was shown that with the progression of CT degeneration, the tensile strength and chordae strain change (Gach et al. 2025). Therefore, understanding the nature and extent of histopathological changes in CT is key to gaining deeper insight into the pathogenesis and natural history of mitral valve disorders.

Chordae tendineae’s role in the course of valvular degeneration has been studied in humans (Baker et al. 1988; Icardo et al. 2013). Research on degenerative mitral valve disease indicates that replacing the normal tissue with proteoglycan-rich pathological tissue compromises the mechanical integrity of the CT. These findings highlight the crucial role of the extracellular matrix composition in maintaining chordal function in humans.

To date, comparable studies of the extracellular matrix in normal and degenerated CTs in dogs have not been reported. The presented work focuses on chordae tendineae in myxomatous mitral valve disease in dogs. The following aspects were considered: (1) histopathological analysis of healthy and degenerated CTs; (2) immunohistochemical assessment of CTs of various proteins and determination of their changes in healthy and degenerated CTs; and (3) correlation between mitral valve lesions and CT in MMVD.

## Materials and methods

### Sample collection and gross examination

Data and sample collection began in January 2021 and ended in January 2023. Hearts were collected from dogs that had been referred to the Department of Pathology for a standard anatomopathological examination and that had died naturally or had been euthanized.

The study included dogs over 8 years of age. The exclusion criteria were the presence of a congenital heart defect, a cardiac disease other than degenerative mitral valve disease, or a neoplastic lesion within the heart. The severity of the mitral valve degenerative changes did not influence inclusion in the study.

Hearts were dissected from the body in the standard way (Janus-Ziółkowska and Bubak 2025); after heart collection, the left atrium and left ventricle were dissected with a longitudinal cut from the cardiac base towards the apex; the right side of the heart was dissected similarly. The heart was carefully flushed with running water and dried. The degree of mitral valve degeneration was subsequently assessed according to the Whitney scale (Whitney 1974), where:

- grade 0 means no visible mitral valve leaflet lesions.
- grade 1 means small nodular lesions located on the free edges of the valve leaflets.
- grade 2 means enlargement of nodule-like lesions.
- grade 3 means connection of large degenerative changes.
- grade 4 means CT involvement of the degenerative process, significant distortion and ballooning of the leaflets.

While lesions grade 1 and 2 are considered mild and do not lead to a symptomatic form of the disease, lesions grade 3 and 4 are defined as moderate and severe, respectively (Aupperle et al. 2009).

**Fig. 1:**
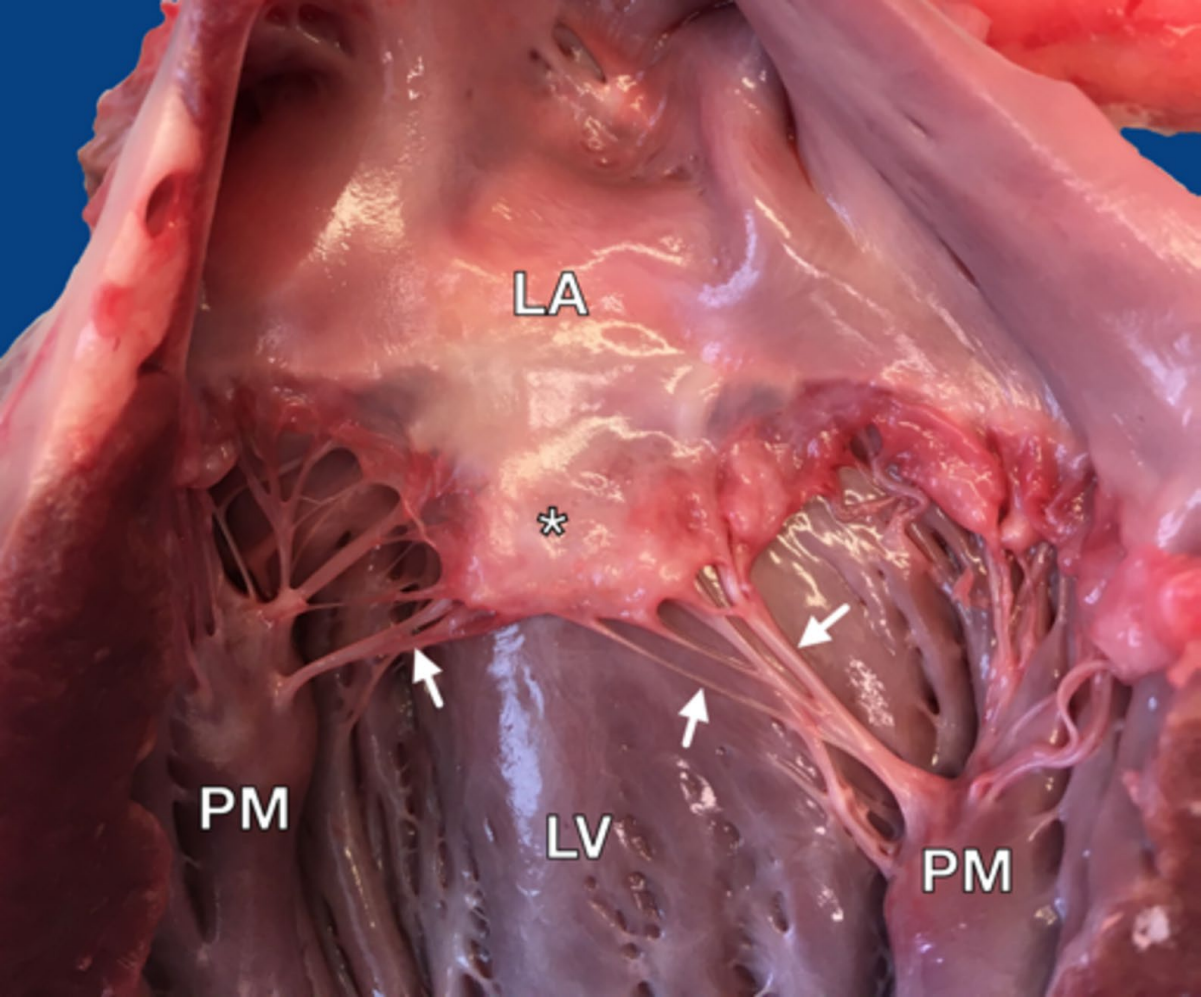
Post-mortem examination of the canine heart. LA – left atrium; LV – left ventricle; * - mitral valve leaflet; PM – papillary muscle; white arrows – CT

After the assessment, the mitral valve with CTs was collected from each heart and immersed in Biolasol fluid (Biochefa, Sosnowiec, Poland). This transplantation fluid was utilised to preserve the tissue’s hydration status while maintaining its biomechanical and structural integrity (Gach et al. 2025). Chordae tendineae were collected and used for biomechanical studies (Gach et al. 2025), and one first-order CT was taken from the anterior mitral valve leaflet from each valve for structural assessment (Fig. 1).

Only first-order CT were taken for study, considering their function and the pathogenesis of mitral regurgitation.

After the collection of the mitral valve with CTs, the heart was cut transversally under the level of the atrioventricular valves, perpendicularly to the cardiac long axis, and the left ventricular internal diameter (LVID) was assessed using a manual Vernier calliper as the distance between the interventricular septum and the left ventricular free wall, which was measured between the papillary muscles on the ventricular cross-section, perpendicular to the ventricular walls. The LVID was subsequently normalised to the animal’s body weight (LVIDn) using the Cornell formula (LVIDn = LVID/(BW)^0.294^; BW = body weight measured in kg) (Cornell et al. 2004).

### Histopathological evaluation

Harvested CTs were immersed in 7% formalin for 24 h for tissue fixation and then inserted into paraffin blocks in a standard manner (Gach et al. 2021). They were cut into 6 μm sections, stained with haematoxylin‒eosin (HE) and picro Sirius red stain and assessed on a semiquantitative scale, as described previously (Gach et al. 2025). The criteria for assessing CT degeneration severity are presented in Table 1.

**Table 1:** Characteristics of different grades of histopathological changes in chordae tendinea (CT) of dogs with myxomatous mitral valve disease (MMVD) evaluated according to histological analysis by Gach et al. 2025

Grade	0	1	2	3
Features	Compact structure, parallel arrangement of fibres, no spaces between fibres	Corrugation of fibre structure and spaces between fibres with diameter smaller than fibre thickness in less than 50% of CT area	Corrugation of fibre structure and spaces between fibres with diameter smaller than fibre thickness in more than 50% of CT area OR spaces between fibres with diameter larger than fibre thickness in less than 30% of CT area	Corrugation of fibre structure with spaces larger than fibre thickness in more than 30% of CT area, chaotic arrangement of fibres, branching structure

Summary of the structural characteristics of CT

The histological analysis was conducted by two independent investigators (JG and IJZ) using a Leica DM500 microscope equipped with a Leica ICC50 W camera (Leica Microsystems, KAWASKA, Poland), and the samples were evaluated at 100x magnification.

### Immunohistochemistry

An immunohistochemical examination allowed us to analyse the difference in protein amount between healthy and diseased CT.

FFPE tissues were freshly cut into 3-µm-thick sections and mounted on Superfrost Plus slides (Menzel Gläser, Germany). Staining was performed on a LEICA BOND-MAX (Leica Biosystems, UK). First, the tissues were deparaffinised (Bond Dewax Solution, Leica Biosystems, UK) and pre-treated with Bond Epitope Retrieval Solution 1 (for collagen I, collagen III, collagen IV, and tenascin C) or Bond Epitope Retrieval Solution 2 (for fibronectin and chondroitin) (Leica Biosystems, UK) for 20 min. The activity of endogenous peroxidase was blocked by Peroxide Block using the BOND Polymer Refine Detection System (Leica Biosystems, UK). In order to evaluate the expression of the studied antigens, antibodies were applied for 15 min at room temperature. The antibody clones and concentrations used are presented in Table 2.

**Table 2:** Characterisation of the antibodies used to detect the proteins in chordae tendineae (CT) of dogs with myxomatous mitral valve disease (MMVD)

Antigen	Clone	Serial number and manufacturer	Concentration
Collagen Type I	5D8-G9	MAB3391 Millipore, Germany	1:75
Collagen Type III	1E7-D7	CSI 007-01-02 Invitrogen, Denmark	1:800
Collagen Type IV	J3-2	SAB 4,200,500 Merck, Germany	1:100
Tenascin C	4 C8MS	MA5-160-86 Invitrogen, Denmark	1:100
Fibronectin	1G10 F9	66042-1-Ig Proteintech, Germany	1:600
Chondroitin	CS-56	MA1-83055 Invitrogen, Denmark	1:250

The antibodies were diluted in Bond Primary Antibody Diluent (Leica Biosystems, UK). Next, the samples were incubated with Post Primary and Polymer using the BOND Polymer Refine Detection System (Leica Biosystems, UK). 3,3’-Diaminobenzidine (DAB chromogen) served as a substrate for the reaction, and all the sections were counterstained with hematoxylin (BOND Polymer Refine Detection System, Leica Biosystems, UK). Negative controls were performed in the absence of the primary antibodies. The canine liver served as a positive control (Baade et al. 2006).

The slides were analysed via an Olympus CX41 microscope (Olympus, Japan). The samples were blinded and examined simultaneously by two investigators, including one veterinary pathologist experienced in immunohistochemical studies. The staining intensity was evaluated semi quantitatively and graded as follows: (-) negative; (+) mild (light-brown); (++) moderate (brown); and (+++) marked (deep-brown). The quantity of each extracellular matrix (ECM) component was assessed as a percentage of the positive area. Owing to the small sample size, the whole CT area was evaluated under 400x magnification, and a mean value for each sample was recorded for each CT.

### Statistical analysis

Statistical analysis was performed using the STATISTICA 13.3 software (TIBCO Software Inc, Kraków, Poland). Data normality was tested using Shapiro-Wilk analysis. Due to the non-normal distribution of data, differences between groups were tested using either Mann-Whitney U analysis or Kruskal-Wallis ANOVA analysis with Dunn post-hoc test. Correlations were tested using Spearman’s correlation analysis. The correlation strength was assessed as poor (0.1 ≤ *r* < 0.3), fair (0.3 ≤ *r* < 0.6), moderate (0.6 ≤ *r* < 0.8), very strong (0.8 ≤ *r* < 1) or perfect (*r* = 1) (Akoglu 2018). Statistical significance was set at *p* ≤ 0.05.

## Results

### Animals

The study included a total of 45 dogs, comprising the following breeds: 22 mixed-breed dogs, 4 American Staffordshire Terriers, 4 Labradors, 3 German Shepherds, 2 French Bulldogs, 2 Cocker Spaniels, 2 Yorkshire Terriers, and one each of the following breeds: Polish Greyhound, Cavalier King Charles Spaniel, Collie Shepherd, Husky, Maltese, and Weimaraner. Out of 45 tested dogs, 23 (51.11%) were male, and 22 (48.89%) were female. The mean age was 11.2 years (range of 8–15 years). The median body weight was 15 kg (range 2.5–38 kg).

### Macroscopic and histological findings

In the study, three (6.67%) mitral valves were classified as unchanged (grade 0, according to Whitney). Among the other cases, 15 valves were classified as grade 1 (33.33%), 16 as grade 2 (35.56%), four as grade 3 (8.89%), and seven as grade 4 (15.55%).

Forty-five first-order CTs were assessed histopathologically. Ten CTs were assessed as healthy (grade 0), and 35 CTs were assessed as degenerated (21 with grade 1, 11 with grade 2, and 3 with grade 3). The CT degeneration grade did not correlate with the mitral valve Whitney degeneration grade (*p* > 0.05; Spearman correlation analysis). A difference in LVIDn was noted between CT degeneration grades (*p* = 0.008; Kruskal-Wallis analysis); specifically, the 2nd -grade CT showed higher LVIDn in diastole values than the 1 st grade CT (*p* = 0.01; Dunn post-hoc test).

### Immunohistochemistry

**Fig. 2:**
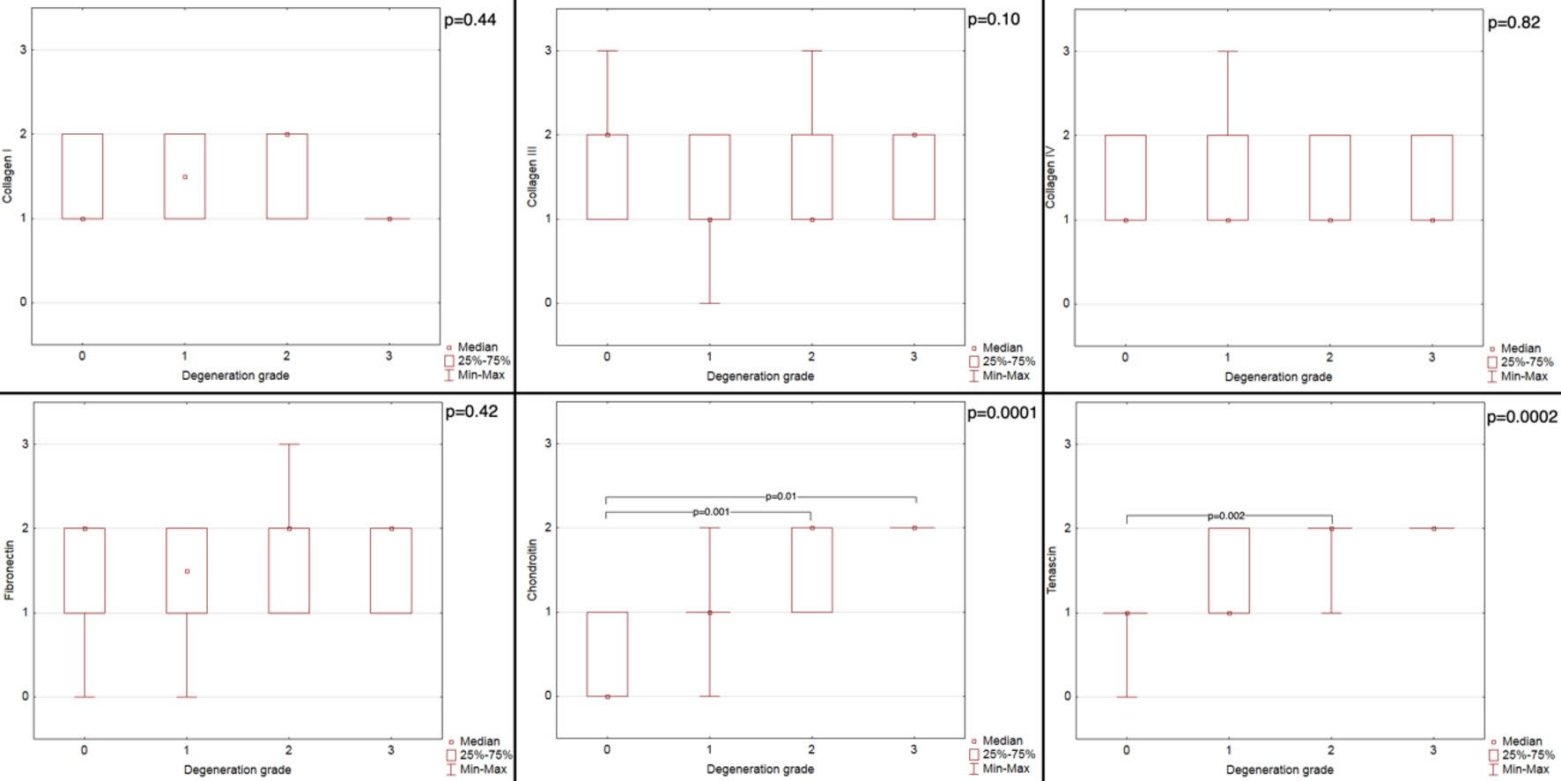
The immunohistochemical reaction intensity for collagen I, collagen III, collagen IV, fibronectin, chondroitin, tenascin in chordae tendineae (CT) of various degeneration grades of dogs with myxomatous mitral valve disease (MMVD)

**Fig. 3:**
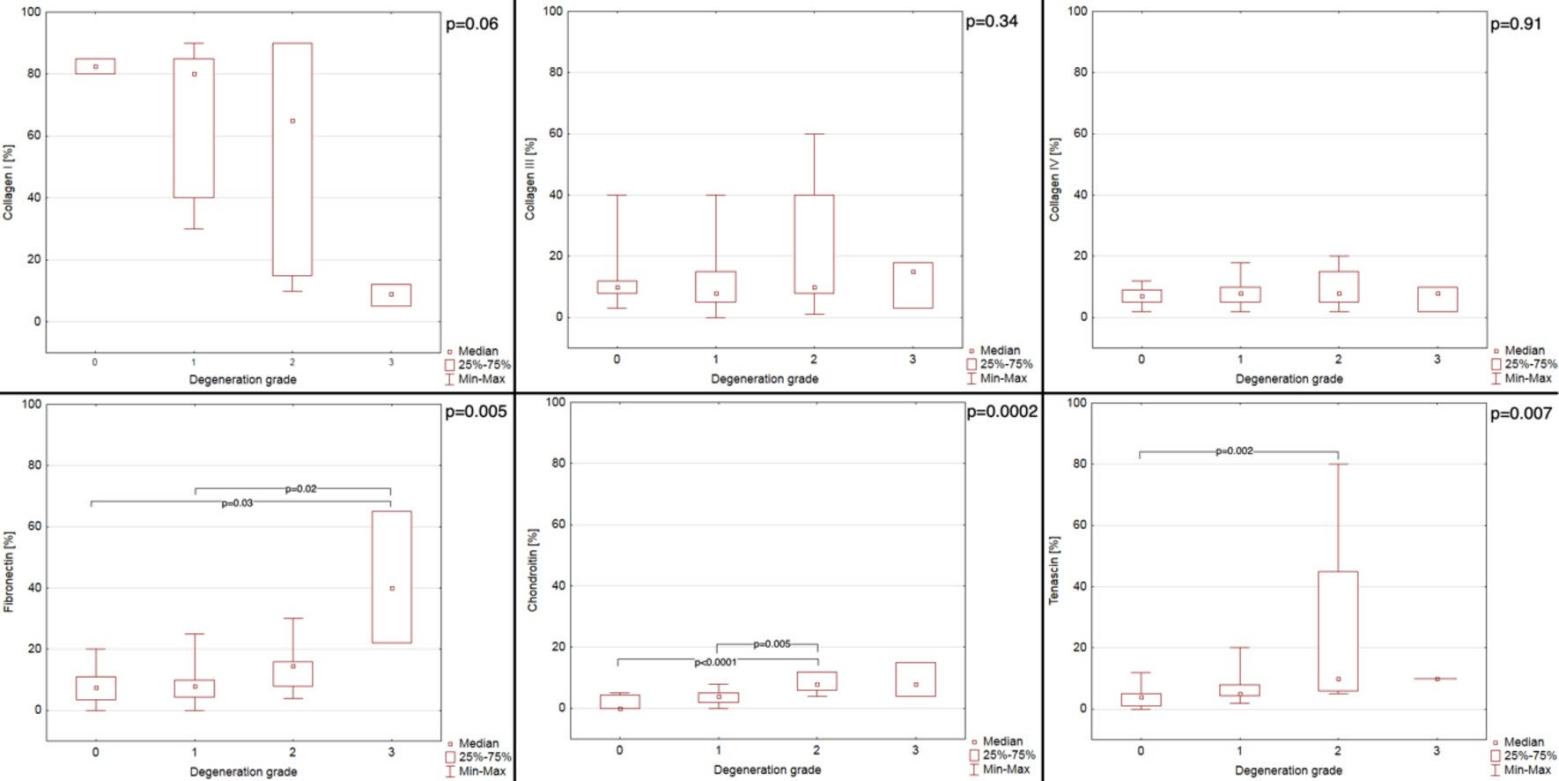
The percentage of positive area in immunohistochemical staining for collagen I, collagen III, collagen IV, fibronectin, chondroitin, tenascin in chordae tendineae (CT) of various degeneration grades of dogs with myxomatous mitral valve disease (MMVD)

**Fig. 4:**
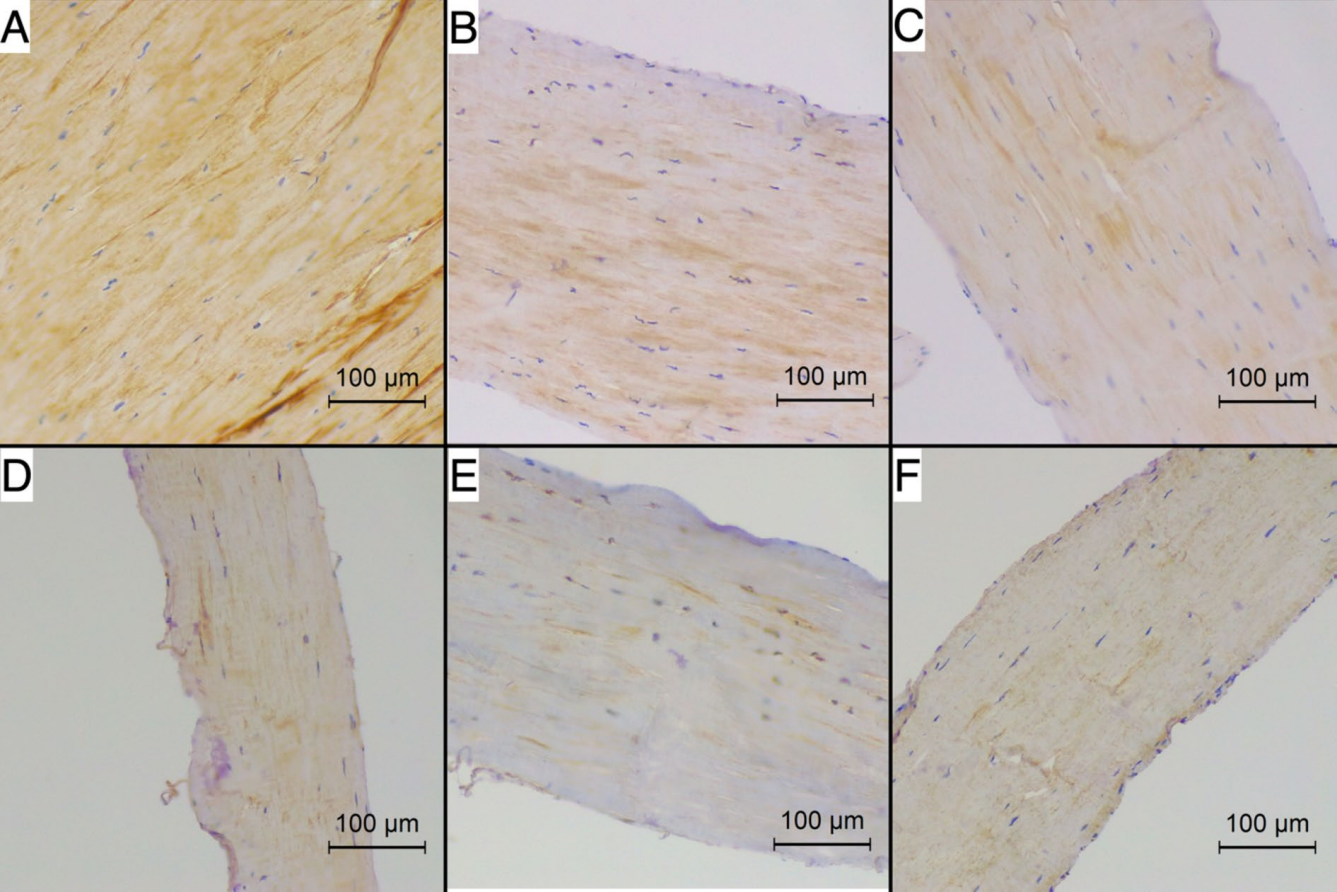
The immunohistochemical reaction for (**A**) collagen I, **B** collagen III, **C** collagen IV, **D** fibronectin, **E** chondroitin, **F** tenascin in healthy chordae tendineae (CT) of dogs with myxomatous mitral valve disease (MMVD). Magnification 200x. Positive reaction is stained brown using 3,3’-diaminobenzidine (DAB) chromogen

**Fig. 5:**
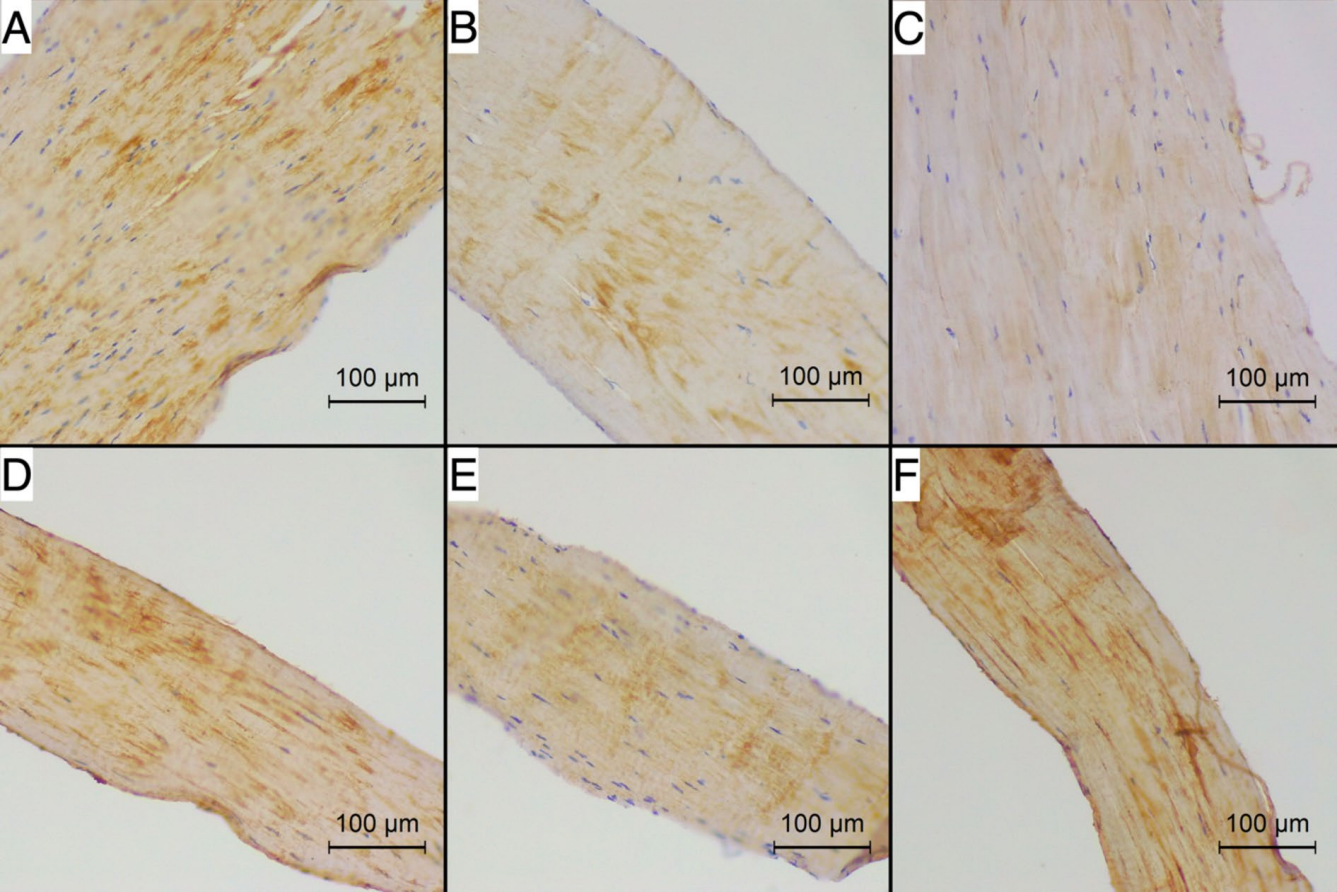
The immunohistochemical reaction for (**A**) collagen I, **B** collagen III, **C** collagen IV, **D** fibronectin, **E** chondroitin, **F** tenascin in degenerated chordae tendineae (CT) of dogs with myxomatous mitral valve disease (MMVD). Magnification 200x. Positive reaction is stained brown using 3,3’-diaminobenzidine (DAB) chromogen

Figures 2 and 3 present the reaction intensity and percentage of positive area in healthy and degenerated CTs for each studied antibody. Figures 4 and 5 present examples of immunohistochemical reactions in healthy and degenerated CTs, respectively.

The analysis of the results revealed that the 0-grade CT showed lower chondroitin intensity than the grade 2 CT (*p* = 0.001; Dunn post-hoc test), and the grade 3 CT (*p* = 0.01; Dunn post-hoc test) and 0-grade CT showed lower tenascin intensity than the grade 2 CT (*p* = 0.002; Dunn post-hoc test).

The analysis of the results revealed the following statistically significant differences: the 0 grade CT showed lower fibronectin percentage of positive area than the grade 3 CT (*p* = 0.03; Dunn post-hoc test); the grade 1 CT showed lower fibronectin percentage of positive area than the grade 3 CT (*p* = 0.02; Dunn post-hoc test); the 0 grade CT showed lower chondroitin positive area percentage than the grade 2 CT (*p* < 0.0001; Dunn post-hoc test); the grade 1 CT showed lower chondroitin positive area percentage than the grade 2 CT (*p* = 0.005; Dunn post-hoc test); the 0 grade CT showed lower tenascin percentage of positive area than the grade 2 CT (*p* = 0.01; Dunn post-hoc test).

#### Collagen I, III, IV

In all the tested preparations, collagen I was globally distributed within the CT structure. Collagen III exhibited a central distribution in 42.5% of the samples and a global distribution in 57.5%. Similarly, collagen IV showed a central distribution in 19% of the samples, whereas 81% of samples exhibited a global distribution. The distribution pattern was not related to the presence of degeneration.

Collagen (any type) did not show any differences in distribution (either intensity of reaction or percentage of positive area) between healthy and degenerated CTs (*p* > 0.05; Mann-Whitney U analysis) or between each grade of CT degeneration (*p* > 0.05; Kruskall-Wallis analysis).

Additionally, collagen I and collagen III showed a fair negative correlation of the percentage of the positive area (*p* < 0.05; *r* = −0.43; Spearman correlation analysis).

#### Fibronectin

Fibronectin was globally distributed in all the samples. Healthy CTs showed no difference in reaction intensity or percentage of positive area (*p* > 0.05; Mann-Whitney U analysis) compared with degenerated CTs. Differences in the percentage of positive area were noted between individual degeneration grades (*p* = 0.005; Kruskal-Wallis analysis). Specifically, the 0-grade CT showed a lower fibronectin percentage of the positive area than 3rd -grade CT (*p* = 0.03; Dunn post-hoc test), and 1 st -grade CT showed a lower fibronectin percentage of the positive area than 3rd -grade CT (*p* = 0.02; Dunn post-hoc test). The percentage of fibronectin positive area fairly correlated with CT degeneration grade (*p* < 0.05; *r* = 0.48; Spearman correlation analysis).

#### Chondroitin sulphate

Chondroitin distribution in the central sample occurred in 6% of the samples tested, and global distribution occurred in 94% of the samples. The distribution pattern was not related to the presence of degeneration.

Compared with degenerated CTs, healthy CTs presented a lower intensity of reaction (*p* = 0.002; Mann‒Whitney U analysis) and percentage of positive area (*p* = 0.008; Mann-Whitney U analysis) than degenerated CTs. Moreover, differences in reaction intensity and percentage of positive area were noted between individual degeneration grades (*p* = 0.0001 and *p* = 0.0002, respectively; Kruskal-Wallis analysis). Specifically, the 0-grade CT showed a lower chondroitin intensity than 2nd grade CT (*p* = 0.001; Dunn post-hoc test), and than 3rd grade CT (*p* = 0.01; Dunn post-hoc test). Also, the 0-grade CT showed a lower chondroitin positive area percentage than 2nd grade CT (*p* < 0.0001; Dunn post-hoc test), and 1 st grade CT showed a lower chondroitin positive area percentage than 2nd grade CT (*p* = 0.005; Dunn post-hoc test). The chondroitin intensity moderately correlated with the CT degeneration grade (*p* < 0.05; *r* = 0.74; Spearman correlation analysis), and the percentage of positive area moderately correlated with the CT degeneration grade (*p* < 0.05; *r* = 0.68; Spearman correlation analysis). Also, fair positive correlation in the percentage of positive area was noted between fibronectin and chondroitin (*p* < 0.05; *r* = 0.48; Spearman correlation analysis) and between tenascin and chondroitin (*p* < 0.05; *r* = 0.38).

#### Tenascin

Tenascin was globally distributed in all the samples.

Healthy CTs showed lower intensity of reaction (*p* = 0.001; Mann-Whitney U analysis) and percentage of positive area (*p* = 0.02; Mann-Whitney U analysis) than degenerated CTs. Moreover, differences in reaction intensity and percentage of positive area were noted between individual degeneration grades (*p* = 0.0002 and *p* = 0.007, respectively; Kruskal-Wallis analysis). Specifically, the 0-grade CT showed a lower tenascin intensity and percentage of positive area than 2nd grade CT (*p* = 0.002 and *p* = 0.01, respectively; Dunn post-hoc test). The tenascin intensity moderately correlated with CT degeneration grade (*p* < 0.05; *r* = 0.70; Spearman correlation analysis), and the percentage of positive area fairly correlated with the CT degeneration grade (*p* < 0.05; *r* = 0.58; Spearman correlation analysis).

## Discussion

The presented study characterised the histopathological and immunohistochemical features of the extracellular matrix components in the CT of normal and degenerated mitral valves affected by canine MMVD. To the author’s knowledge, no studies have assessed the collagen I, III, IV, fibronectin, tenascin, and chondroitin contents of canine CT. Aupperle et al. assessed similar ECM components in canine MMVD, but focused on the mitral valve leaflets rather than the CT (Aupperle et al. 2009).

Degenerative CT changes are common in older dogs. Mitral valve disease, prevalent in small- and medium-sized breeds, is also age-related, with its risk increasing over time (Mattin et al. 2015). Our study further highlights the widespread occurrence of mitral valve degeneration and CT degeneration in older dogs, including small, medium, and large breeds. Even if mitral valve leaflet lesions are not visible on anatomopathological examination (Whitney grade 0), degenerative changes in some CTs are present, resembling those observed in clearly affected mitral valves (Whitney grade 2, 3, or 4). Valvular and CT degenerative lesions are unevenly distributed and are not significantly correlated, indicating the partial independence of CT degeneration from mitral valve disease (Fox 2012; Icardo et al. 2013). This may explain cases where CT rupture occurs in mild stages of MMVD. A retrospective evaluation of cases of CT rupture in dogs (Serres et al. 2007) showed that 24.6% of the dogs were asymptomatic at the time of the episode. CT rupture may be associated with the life-threatening phenomenon of acute pulmonary oedema. Still, in some cases, it may demonstrate reverse remodelling, allowing chronic heart failure therapy to be discontinued.

Studies in human CTs indicate that the increase in collagen and GAGs and fragmentation of elastic fibres in CTs are affected by degeneration (Lis et al. 1987; Fornes et al. 1999; Grande-Allen et al. 2003). In our study, noticeable changes within the ECM were observed. As degenerative changes in CT progress, the expression of fibronectin, chondroitin, and tenascin increase. These alterations reflect an active degenerative process. However, our data did not reveal statistically significant differences in collagen types I, III, and IV levels. It is worth noting a trend: in healthy CT (grade 0), collagen type I is predominant, ensuring optimal mechanical properties. As degenerative disease advances, the amount of collagen type I in CT decreases. These modifications result in mechanically weakened CT, making it more susceptible to rupture and chronic dysfunction. In summary, as CT degeneration progresses, structural remodelling occurs, characterised by the increasing replacement of collagen I with collagen III, tenascin, fibronectin, and chondroitin sulphate.

As mentioned previously, older animals are more susceptible to mitral valve and CT degeneration. Therefore, one cannot rule out the influence of ageing on the structure of the tissues. Observations on human CT indicate that the ageing process of connective tissue is associated with progressive disorganisation of the collagen core, confirming our data on CT degeneration (Millington-Sanders et al. 1998).

Finally, we would like to underscore the impact of left ventricular remodelling on CT lesions. In our study, CTs assessed as the 2nd grade had higher LVIDn values than the 1 st grade CTs. Increased left ventricular volume results in increased tension on CT, which may also stimulate molecular remodelling (Nazari et al. 2000; Madhurapantula et al. 2020). One experimental study on CT in sheep (Dal-Bianco et al. 2009) revealed that stretched CTs have reduced collagen alignment and density. Research suggests that prolonged mechanical stress on CT, induced by increased left ventricular dimensions, may activate molecular pathways and stimulate progressive extracellular matrix remodelling.

An additional value of these results may be the provision of new knowledge to veterinary cardiac surgery and mitral valve replacement procedures. Depending on the technique used, i.e., if primary CTs are left, the aetiology of the disease is uncorrected. Each CT is a potential target for degenerative disease, making it possible for complications to develop years after surgery in the form of rupture of the native CT and lead to mitral regurgitation and, ultimately, progression of the disease to heart failure (Flameng et al. 2008; Mizuno et al. 2013). The obtained data align with findings from human studies, suggesting that partial CT correction may not represent the most effective long-term strategy and that a more aggressive approach could be warranted (Lindmark et al. 2009; Icardo et al. 2013).

The primary limitation of this study is the absence of an echocardiographic assessment to confirm MMVD and determine its precise stage according to the ACVIM classification. Additionally, only three dogs were classified as grade 3 based on histopathological evaluation, resulting in a small sample size for this category. Finally, only a single CT sample was collected from each dog, as the remaining valve apparatus was allocated to other studies published elsewhere (Gach et al. 2025).

In conclusion, the healthy CT of canine mitral valves exhibit a distinct distribution pattern of various matrix components that changes with the progression of degeneration. To our knowledge, this is the first description of collagen I, III and IV, fibronectin, chondroitin, and tenascin in CT in dogs. The disorganisation of the extracellular matrix in degenerating chordae, as analysed through immunohistochemical methods, provides novel insights into the pathophysiology of this disease. Our findings suggest that affected CTs exhibit increased matrix synthesis and degradation, disrupting the organised collagen structure and altering their structural and mechanical properties. From a mechanistic perspective, these changes contribute to CT rupture. Moreover, we found that CT degenerative changes are independent of mitral valve degeneration, suggesting a possibly more complex relationship and a need for further focus on CTs in dogs with myxomatous mitral valve disease.

## Data Availability

The majority of the data supporting the findings of this study are included within the manuscript. Other relevant datasets can be obtained from the corresponding author upon reasonable request.

## Abbreviations

CT: Chordae tendineae
ECM: Extracellular matrix
GAG: Glycosaminoglycan
HE: Haematoxylin–eosin
MMVD: Myxomatous mitral valve disease
